# The characteristics of intratumoral microbial community reflect the development of lung adenocarcinoma

**DOI:** 10.3389/fmicb.2024.1353940

**Published:** 2024-04-24

**Authors:** Yanfang Su, Shiyu Li, Die Sang, Yurong Zhang

**Affiliations:** Department of Medical Oncology, Beijing Chaoyang District Sanhuan Cancer Hospital, Beijing, China

**Keywords:** lung adenocarcinoma, microbiome, tumor stage, random forest, co-occurrence networks

## Abstract

**Introduction:**

An increasing number of studies have demonstrated the pivotal role of microbiota changes in the onset, progression, diagnosis, treatment, and prognosis of lung adenocarcinoma (LUAD). However, a comprehensive analysis of intratumoral microbiome variation across distinct LUAD stages has not been performed. The aim of this study was to identify the microbial markers that significantly vary during tumor stage of LUAD.

**Methods:**

Here, we used the cancer genome atlas (TCGA) database to comprehensively compare and analyze the differences in microbial composition between 267 patients with early and 224 patients with advanced LUAD. In order to determine the best biomarkers, we used the random forest (RF) model and found that the microbial markers have a certain ability in predicting the stage of LUAD.

**Results:**

We found that there were certain differences in the microbiome of patients with LUAD at different stages, especially in the tumor tissues of patients with advanced LUAD, whose co-abundance network was significantly more complex. We also found that five bacterial biomarkers (*Pseudoalteromonas*, *Luteibacter*, *Caldicellulosiruptor*, *Loktanella*, and *Serratia*) were correlated with LUAD stage, among which *Pseudoalteromonas*, *Luteibacter*, *Caldicellulosiruptor*, and *Serratia* were significantly overexpressed in patients with advanced LUAD. In particular, after integrating the biomarkers of mRNA, we achieved an area under the curve (AUC) of 0.70.

**Discussion:**

Our study revealed the microbial profile of patients with LUAD and the intrinsic pathogenic mechanism between the microbiome and the disease, and established a multi-omics model to determine LUAD tumor stage.

## 1 Introduction

Lung adenocarcinoma (LUAD) represents a form of non-small cell lung cancer and stands as one of the most lethal tumors globally (Chen et al., 2017; Li et al., 2019). Despite advancements in the diagnosis and treatment of lung diseases, patients with LUAD continue to experience high mortality and a poor prognosis, with an average 5-year survival rate of only 15% (Riihimäki et al., 2014; Song et al., 2020; Sung et al., 2021; Yang et al., 2021). One major contributing factor to the high mortality rate of lung cancer is the asymptomatic nature of most early-stage patients, resulting in late-stage diagnosis for confirmed cases (Nasim et al., 2019; Mo et al., 2020). Additionally, lung cancer patients commonly experience recurrence and metastasis following surgical resection (Popper, 2016). The treatment options and survival rates vary significantly between early and late-stage patients (Oudkerk et al., 2021). Hence, the precise identification of biomarkers associated with the early stage of LUAD may offer novel insights into tumorigenesis and early preventive measures, aiding doctors in evaluating patients’ status and adjusting treatment strategies (Miao et al., 2021; Yang et al., 2021).

Some studies have shown that some mRNA molecules are related to the progress of patients with LUAD (Chen et al., 2019). Dong et al. (2021) found that *ZLC5* was up-regulated in lung cancer patients, and its high expression predicted a shorter overall survival (*p* = 0.007), and as an independent prognostic marker of lung cancer, HR = 2.892; 95% (Yang et al., 2021) CI: 1.297–6.449; *p* = 0.009; Zhang et al. (2019) found that nine mRNA genes (*HMMR, B4GALT1, SLC16A3, ANGPTL4, EXT1, GPC1, RBCK1, SOD1 and AGRN*) were associated with the overall survival rate of lung cancer patients. Through multivariate Cox regression analysis, the prognostic ability of nine gene characteristics were higher than that of clinical information; Xin et al. (2019) found that the mRNA levels of *COX-2, cPLA2, COX-1, mPGES, PGE2 and PGI2* in lung cancer patients were significantly higher than those in healthy people, especially in patients with high expression of *mPGES* and *PGI2*. The 5-year survival rate was lower than that in patients with low expression of *mPGES* and *PGI2*, and it was statistically significant for the prognosis of lung cancer. Although some studies have identified some molecular markers for predicting lung cancer, it is still difficult to achieve high-precision prediction due to the lack of information in a single omics (Shi et al., 2022). Simultaneously, there remains a dearth of pertinent research and predictive assessment on the status of patients’ tissue microbiome.

Recent advancements in microbiome research have revealed associations between various diseases and alterations in the gut microbiome (Rezasoltani et al., 2017; Fan et al., 2018; Sepich-Poore et al., 2021; Zhou et al., 2023). Among them, the most typical example is the correlation between *Helicobacter pylori (H.P)* infection and gastric cancer (El-Omar et al., 2003). *Helicobacter pylori* infection will lead to methylation of tumor related gene CpG island in gastric epithelial cells (Kim et al., 2021; Wang et al., 2021). It may also lead to peptic ulcer, or even the generation and development of gastric cancer (Suez et al., 2018) by inhibiting cell apoptosis. The change of *Fusobacterium nucleatum* is related to colorectal cancer (Cheng et al., 2019a). The study revealed an intriguing finding that, in addition to the gut microbiota, a considerable number of microorganisms exist in tumor tissues, potentially playing a significant role in cancer development (Wang et al., 2021). Nevertheless, the potential relationship between the tissue microbiome and patients with LUAD at various stages remains unclear.

Evidently, surgery, radiotherapy, chemotherapy, targeted therapy, and other conventional methods can aid in reducing the mortality and incidence rates in LUAD patients. However, challenges persist, including high costs and inherent risks (Bray et al., 2018). Hence, researchers are dedicated to exploring supplementary strategies for diagnosing LUAD at various stages, particularly investigating the potential use of microbiome as biomarkers (Yuan et al., 2022). Studies have shown that there was a significant relationship between *Mycobacterium tuberculosis* and lung cancer (Robinson and Smyth, 2007). *Ruminococcus, Eubacterium* and adolescent *Bifidobacterium* were enriched in lung cancer patients. In particular, *Enterococcus* and *Pasteurella* were significantly overexpressed in patients with advanced lung cancer. In addition, Zheng et al. (2020) analyzed 13 microbiome as biomarkers, and the prediction accuracy reached AUC = 97.6% in patients with early lung cancer and AUC = 76.4% in independent validation cohort. Therefore, the article delves into the microbiome profiles of patients with LUAD at various stages and investigates the potential mechanisms of their interactions.

In our study, we collected a discovery cohort of 267 patients with early LUAD and 224 patients with advanced LUAD, and comprehensively analyzed their tissue microbiome and transcriptome profiles. The main purpose of this study is to find the microbial profiles of LUAD patients and identify multi omics features that can distinguish patients with early and advanced LUAD.

## 2 Methods and materials

### 2.1 Patients’ cohort and data preparation

The host transcriptome, tumor microbiome data, and metadata in this study were all from TCGA public database. The tumor microbiome data of patients with LUAD was derived from the recleaning of sequencing data in TCGA by Rob knight’s group (Poore et al., 2020). We chose to use patient tissue microbial data obtained by RNA sequencing (RNA-seq). According to the pathological stage after the initial diagnosis, the samples were divided into two categories. We defined the patients with pathological stage I as “early” and the patients with stages II–IV as “advanced.” A total of 491 samples were downloaded, including 267 “early” and 224 “advanced.” Each sample has corresponding clinical information such as age, sex, tumor node metastasis classification (TNM) stage and host gene expression.

### 2.2 Statistical analysis

R software (version 4.3.1) was used for statistical analysis. Considering that microbial data are sparse and non-normally distributed, we employed non parametric tests for correlation statistics. Wilcoxon rank-sum test was used to determine the association between different clinical characteristics and stages. T-test was conducted to identify the bacterial biomarkers whose abundance was significant different between patients with early and advanced stage. A *p*-value less than 0.05 was considered as statistical significance.

### 2.3 Microbial diversity analysis

The alpha diversity was measured by the Shannon index, which was calculated by function “diversity” in “vegan” package in R. Principal coordinate analysis (PCoA) was performed with the “vegan” package in R to analyze the differences in intratumoral microbial communities between groups, and the permutational multivariate analysis of variance (PERMANOVA) was used to conduct the statistical test. Wilcoxon rank-sum test was used to test the difference in microbial diversity between the two groups.

### 2.4 Microbial network analysis

To study the association between microbiome, we used spearman rank correlation to construct microbial interaction networks at the genus level. The “hmisc” package in R language was used to calculate the correlation and *p*-value. When the correlation coefficient between bacterial populations is >0.7 and the *p*-value is <0.001, it is considered to have significant correlation. Network visualization was performed using Gephi (version 0.9.6). In our network, node stands for genus, and edge stands for spearman’s rank correlation. Degree is the number of edges on each node. The higher the degree of a node, the more points it is connected, and the more critical it is. The clustering coefficient indicates the degree of connection between a node and its adjacent nodes.

### 2.5 Transcriptome analysis

The R package “Deseq2” was used to identify differentially expressed genes (DEGs) of mRNA (Costa-Silva et al., 2017; Cheng et al., 2019b). We chose to use *p*-value < 0.0001 and log2foldchange (LFC) > 1 to screen the up-regulated genes, and when *p*-value < 0.0001 and LFC < −1, it was defined as the down-regulated genes. Finally, we used | log2 (foldchange) | ≥ 1 and adjusted *p*-value < 0.05 to obtain. The volcano map showed the distribution of the gene map of patients with LUAD. The R package “pheatmap” was used to visualize significantly different genes. Gene Ontology (GO) enrichment analysis was performed using the “clusterprofiler” package in R software. The enrichment paths of DEGs were visualized by the “ggplot2” package.

### 2.6 Model construction and evaluation

Construction and evaluation of lung cancer diagnosis model based on multi omics features. Based on the microbial features obtained by different feature screening methods and transcriptome features, the lung diagnosis of potential markers was comprehensively analyzed, mainly including cross validation model construction and model evaluation. We label patients with early LUAD as “0” and patients with advanced LUAD as “1,” which translates our study into a binary classification of machine learning. Previous studies showed that random forest performed well on similar data types (Rigatti, 2017; Cheng et al., 2018; Li et al., 2019, 2021), thus, random forest was used to build the model in our study. Models were trained using data from the host genes, microbes and combination of the two omics. Classification and Regression Tree (CART) and Bagging technique are used in random forest algorithm. CART can be applied to both classification and regression. The minimum Gini index is used as the segmentation rule when CART is used as classification tree. The GridSearchCV was used to adjust three parameters, including n_estimators, max_depth, and max_features, and adjust the parameters min_samples_split and min_sample_leaf.

## 3 Results

### 3.1 Gender was significantly correlated with pathological stage of LUAD patients

**Figure 1:**
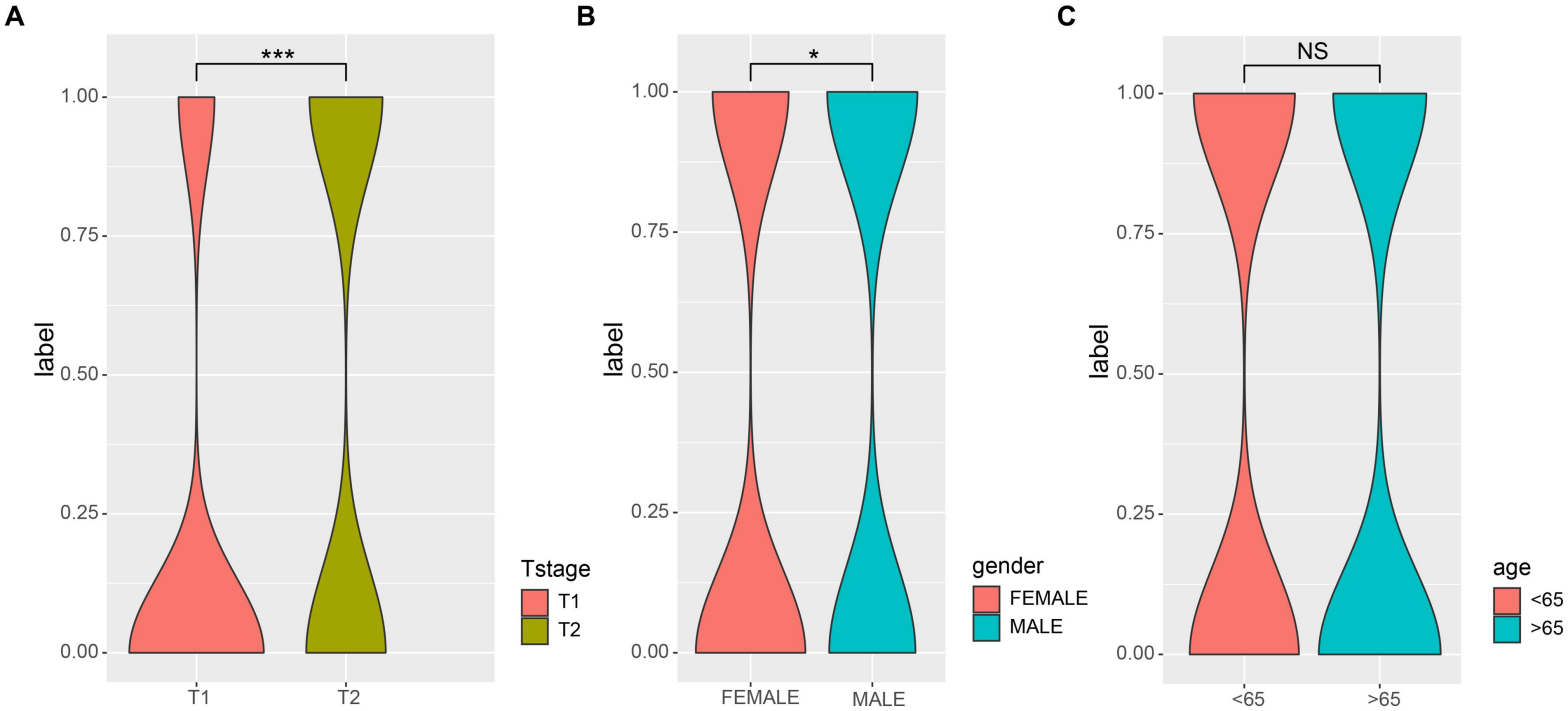
The correlations of different clinical characterization with patients of early and late. **(A)** The depth of tumor and the range of adjacent tissue involvement increased significantly in patients with advanced LUAD; **(B)** There was a significant correlation between the gender and LUAD stage; **(C)** There was no significant difference in age (>65) among patients with LUAD. Wilcoxon test was used to compare between two groups. 0, early; 1, advanced; ^*^p-value < 0.05, ^***^p-value < 0.001.

First, we examined the association between LUAD stage and clinical parameters. The correlation between clinical parameters and LUAD stage was shown in Table 1. There were significant differences in T stage (Figure 1A, *P* < 0.001) and gender (Figure 1B, *P* < 0.05) between patients with early and advanced LUAD. In addition, there was no significant difference in age, N stage, and M stage between patients with early and advanced LUAD (Figure 1C; Table 1). These results proved that there was a significant difference in gender between patients with early and advanced LUAD. It should be noted that the higher proportion of men with advanced LUAD may be due to higher smoking rates. The specific information of all patients was shown in Table 1.

**Table 1:** Clinical information.

Parameters	Early (*n* = 267) 0	Advanced (*n* = 224) 1	*p-value*
Gender (M/F)	110/157	115/109	*
Age (avg years)	65.89	64.57	NS
N0/N1/N2/N3/NX/unknown	257/2/0/0/7/1	58/90/70/2/4/0	NS
M0/MX/unknown	179/85/3	23/2/12	NS
T1/T2/T3/T4/unknown	131/136/0/0/0	3/7/24/1/2	***

Early, patients with stage I; Advanced, patients with stages II–IV; NS, not significant; ^*^p-value < 0.05, ^***^p-value < 0.001.

### 3.2 There were differences in the microbial profiles of patients with LUAD between the two groups

**Figure 2:**
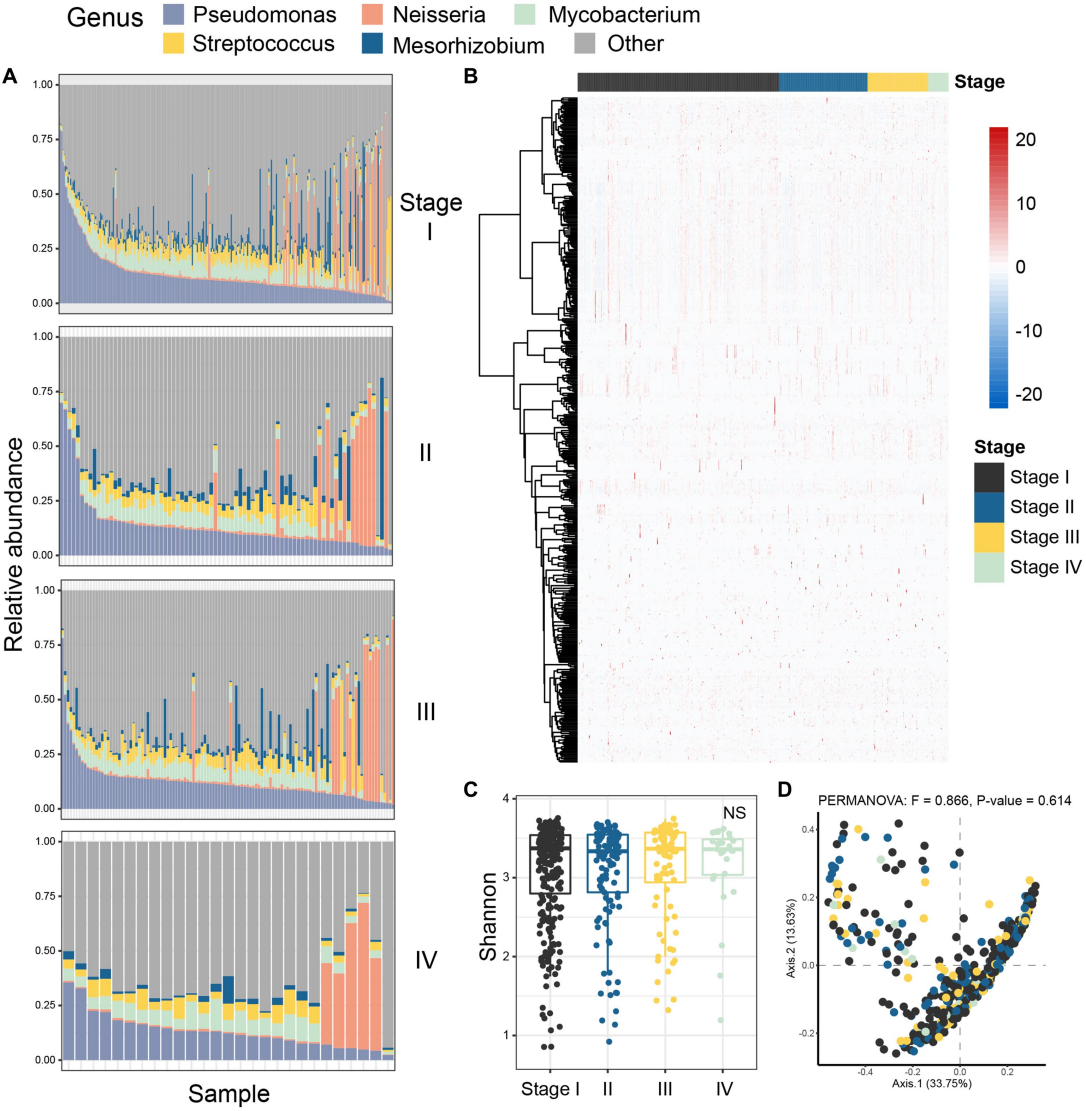
Colony analysis of microbiome data in patients with lung adenocarcinoma. **(A)** The composition of microorganisms in patients at four different stages (stage I, stage II, stage III, stage IV) at the genus level. The stacked diagram only shows the genus of top 5, and the rare genus was summarized as other. **(B)** The heat map showed the Spearman correlation of all samples in four stages; **(C)** Shannon index was used to measure the alpha diversity among different staging cohorts of patients with LUAD; **(D)** PCoA was performed on samples from all four cohorts based on Bray Curtis distance. The results showed that there was no difference in microbial composition between patients in the four stages (*p*-value = 0.614). The beta diversity *p-*value based on Bray Curtis distance was calculated by 999 permutations (two-sided test); PCoA, Principal coordinate analysis; Black, Stage I; Blue, Stage II; Yellow, Stage III; Green, Stage IV.

**Figure 3:**
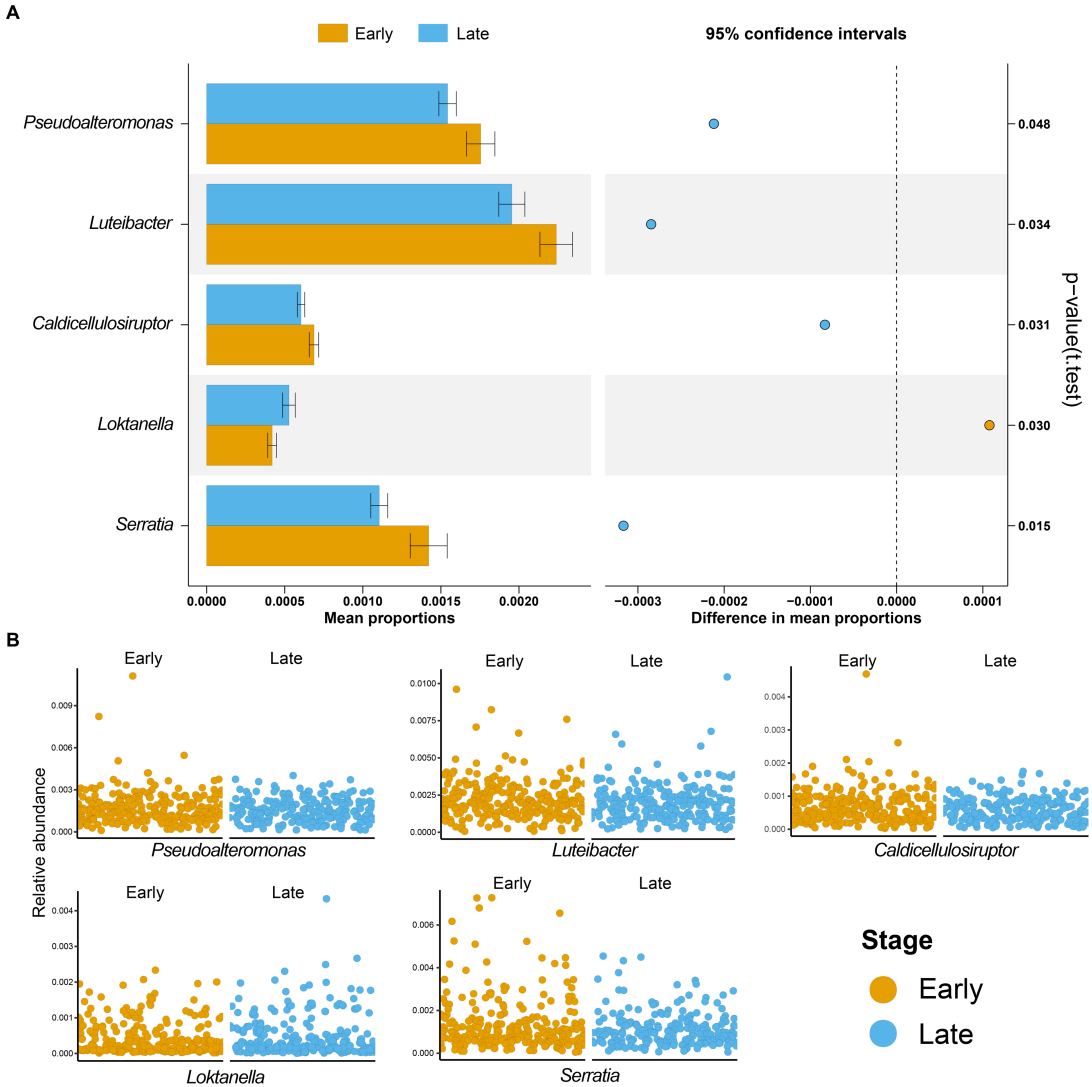
Microbial biomarkers of lung adenocarcinoma. **(A)** Five microbial markers were identified by t-test, including *Pseudoalteromonas, Luteibacter, Caldicellulosiruptor, Loktanella,* and *Serratia.* The bar graph is the mean proportions of the five markers, and the dot graph is 95% confidence intervals between the two groups, p-value showed that there was a significant difference between patients with early and advanced LUAD; **(B)** The relative abundances of five microbial markers in the two groups were shown. Yellow, patients with early LUAD; Blue, patients with advanced LUAD.

Next, we characterized the intratumoral microbiome profiles for all patients (Figure 2). We detected that genus *Pseudomonas*, *Streptococcus*, *Mycobacterium*, *Neisseria*, and *Mesorhizobium* constituted the dominant content of intratumoral microbial community in lung. Specifically, *Pseudomonas* was the genus with the highest relative abundance in lung tumors. At the same time, we also evaluated the alpha diversity level of the samples. Shannon index showed that there was no significant difference in microbial diversity among patients at different stages (Figure 2C), while PCoA (*p* = 0.614) also showed that there was no significant difference in bacterial communities among the four stages (Figure 2D). However, through statistical test, we still identified five bacteria whose abundances were significantly different between the early and late patients (Figure 3; *p* < 0.05), which were used as the microbial markers for downstream analysis.

### 3.3 Changes of bacterial co-abundance network in LUAD patients at different stages

**Figure 4:**
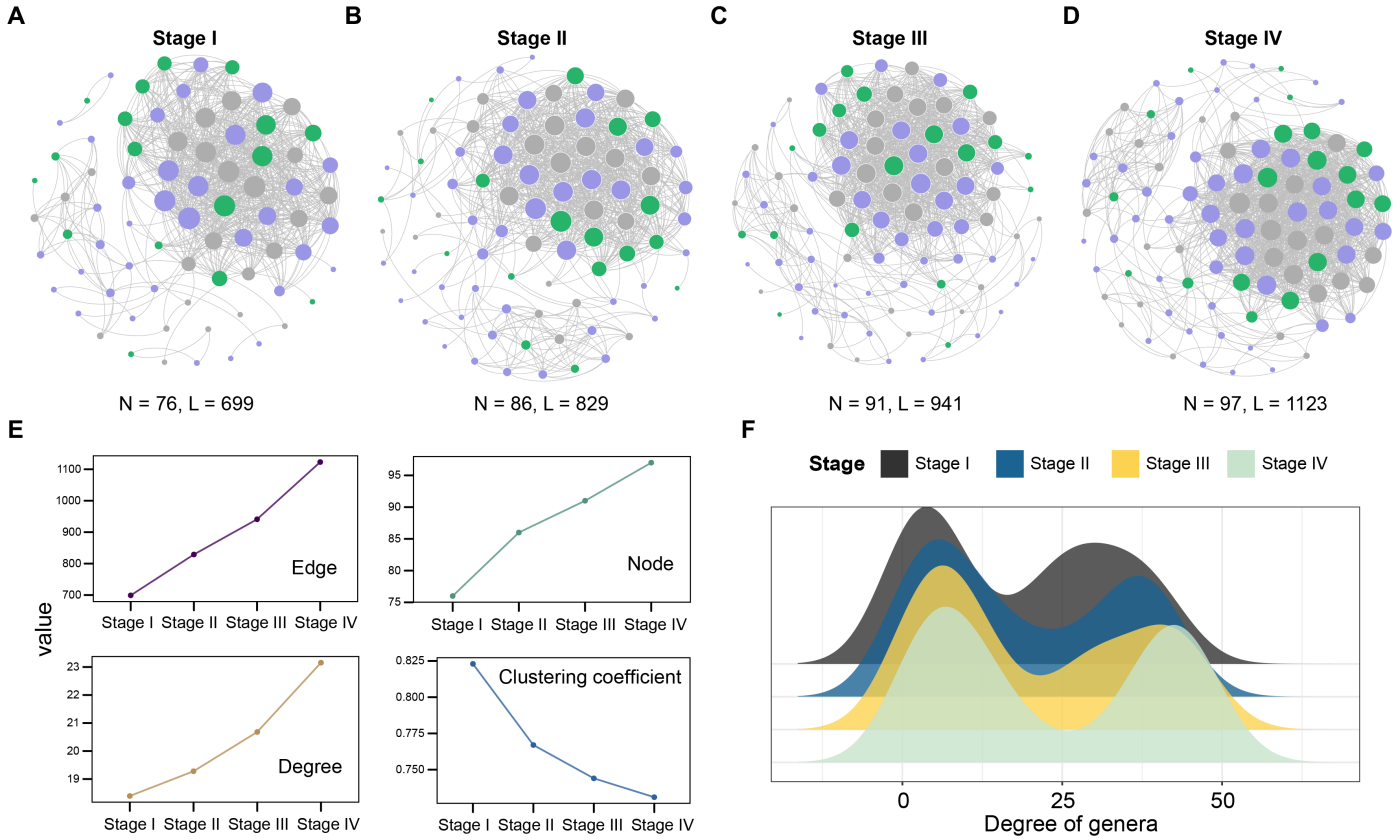
Network analysis revealed the co-abundance correlation of microbial communities in patients with LUAD at different stages. **(A)** Stage I; **(B)** Stage II; **(C)** Stage III; **(D)** Stage IV common abundance network of all samples. The color of the nodes indicate that the flora comes from *Firmicutes* (purple), *Proteobacteria* (green), and other phylum levels (gray). The size of the circle represents the size of the correlation. The larger the circle, the greater the correlation, only significant absolute correlations (r > 0.7, significant at *p*-value < 0.001) were shown; **(E)** The line chart showed the change of the correlation coefficient of the patient network in the four stages. Nodes represent the genus, while edges represent the rank correlation of Spearman, degree is the number of edges on each node. The higher the degree of the node, the more points connecting it, and the more critical it is. The clustering coefficient indicates the degree of connection between a node and its neighboring nodes; **(F)** The degree of microbial genus in patients at four different stages. Black, Stage I; Blue, Stage II; Yellow, Stage III; Light green, Stage IV.

Previous studies have shown that there are differences in the microbial composition of patients with LUAD at different stages. In order to gain insight into the potential interactions between bacteria at each stage, we conducted a co-abundance association analysis based on their abundance. In summary, the complexity of patient network varies greatly in different stages. With the development of the patient’s condition, especially in stage IV, the complexity of the microbial community network reaches the maximum. The co-abundance networks of stage IV patients (97 species and 1,123 associations, Figure 4D) were more complex than that of stage I (76 species and 699 associations, Figures 4A–C). At the same time, the degree was significantly increased (Figure 4E), which reflected that the association between the intratumoral microbes was closer in the patients with advanced LUAD. In addition, we detected that the degree of genera in microbial co-abundance network gradually increased from stage I to stage IV (Figure 4F). These results suggest that disease progression in patients with LUAD is accompanied by changes in the pattern of intratumoral microbial interactions.

### 3.4 The mRNA gene expression profiles of lung cancer patients at different stages were significantly different

**Figure 5:**
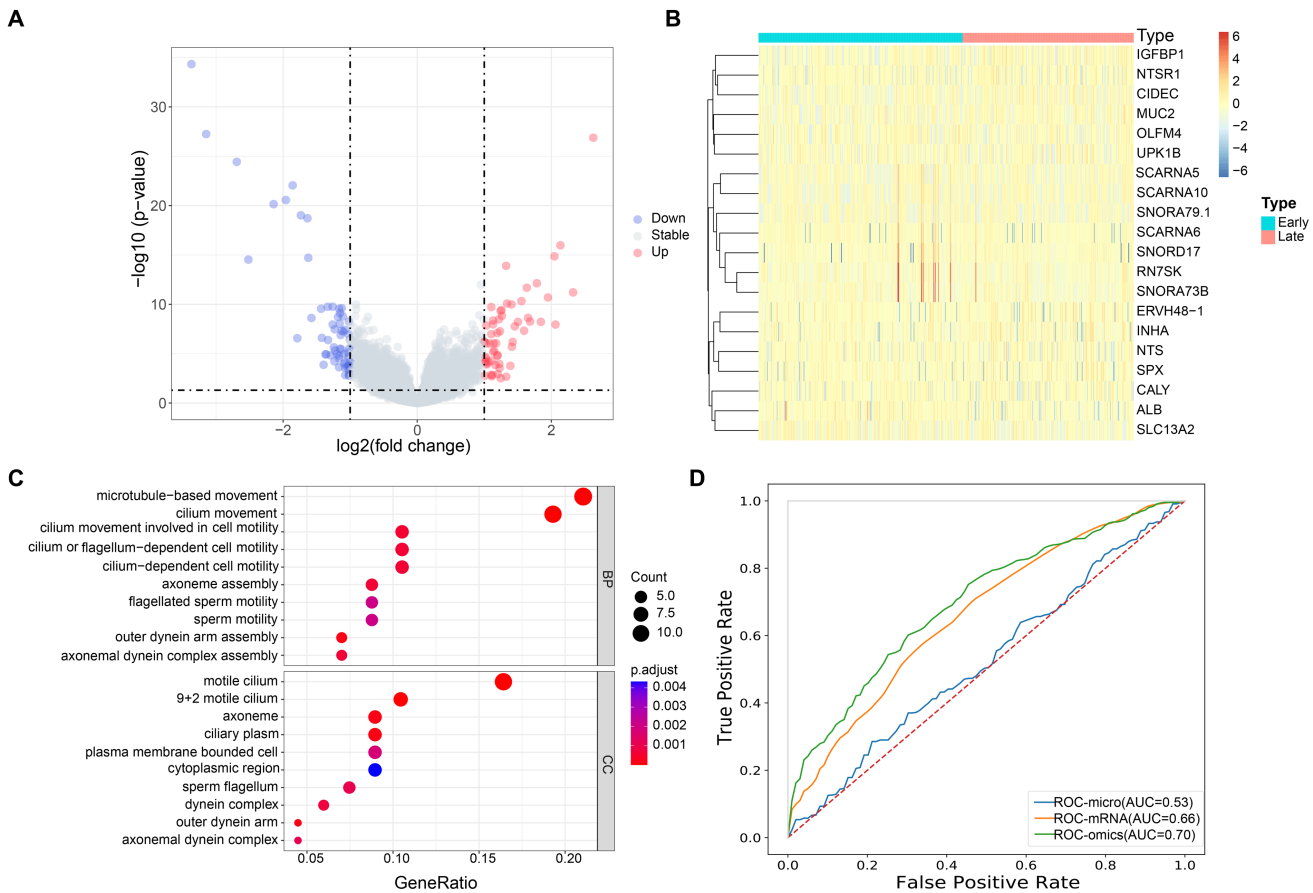
Transcriptome profiles of patients with early and advanced LUAD. **(A)** The volcano map showed the distribution of all gene expression, blue, downregulated genes; red, upregulated genes; gray, no significant difference genes; **(B)** The heat map of the DEGs of mRNA between the early and late group, the x-axis is the sample of two groups, and the y-axis is the top 20 expressions with significant differences screened by DEseq2; **(C)** GO enrichment analysis, the names of the top 10 pathways enriched on the Y axis, and the proportion of enriched genes in the corresponding pathways on the X axis; **(D)** AUC accuracy of staging prediction of LUAD patients by different omics.

Next, we analyzed the host gene expression profiles of patients with early and advanced LUAD. Among 491 samples, there were 122 genes with significant differences in mRNA expression, of which 60 genes were significantly up-regulated and 62 were down-regulated (Figure 5A). The expression of these 122 genes was significantly different between the two groups, and we used the top 20 for display (Figure 5B). In addition, we also found that the up-regulated genes were significantly enriched in patients with early LUAD, especially SNORD17, RN7SK and SNORA73B. Through the GO enrichment analysis of differential genes, we found that in the biological process (BP) category, significant differential genes were mainly enriched in microtubule-based movement and cilia movement involved in cell mobility. For the cell component (CC) category, significantly different genes were mainly clustered in plasma membrane bounded cell and axonal dynein complex (Figure 5C).

### 3.5 Prediction of LUAD staging by multi omics

Based on the previous studies, we infer that the differential microbiome may have a certain predictive ability for the stage of patients with LUAD. To this end, we constructed a machine learning model to classify patients. We evaluated the predictive power of different omics for patients with LUAD. Random forest five-fold cross validation showed (Figure 5D) that mRNA had the strongest ability to predict the stage of patients with pancreatic cancer, with AUC = 0.66. At the same time, we chose to use the microbial markers and mRNA differential genes obtained earlier for joint prediction, with AUC = 0.70. Compared with single omics, the prediction accuracy of the model was significantly improved. This showed that the bacterial markers we screened have a certain predictive ability for the staging of patients with LUAD, and can make up for the shortcomings of single omics.

## 4 Discussion

LUAD is a common malignant tumor, and one of the prominent reasons for its high mortality is that most patients are only diagnosed in the late stage of cancer (Nasim et al., 2019; He et al., 2020). Although, surgery, chemotherapy and radiotherapy are helpful to the treatment of patients, effective early detection and detailed pathogenesis are more conducive to reducing the mortality of patients with LUAD. Therefore, the early diagnosis of patients with LUAD is particularly important, which urgently requires the identification of new specific biomarkers (Nooreldeen and Bach, 2021; He et al., 2022). We carried out an innovative analysis of the tissue microbial atlas of early and late patients. Although their alpha and beta diversity was similar, the co-abundance network of late patients was more complex. Finally, we identified five bacteria that can distinguish early and late patients. Among them, four specific microbial markers (*Pseudoalteromonas, Luteibacter, Caldicellulosiruptor, and Serratia*) were also significantly overexpressed in patients with advanced LUAD. In addition, in order to identify the best marker panel, we constructed a machine learning classification model to predict the stage of patients with LUAD. The results showed that tissue microbial biomarkers have a certain predictive ability for the staging of patients with LUAD, and can make up for the shortcomings of other single omics. Microbial markers combined with transcriptome DEGs can predict the stage of patients with LUAD, and the AUC can reach 0.70.

It has been reported that human microbiome especially *Helicobacter pylori, Proteobacteria, Bacteroidetes fragilis* and *Fusobacterium nucleatum*, have been proved to be related to the occurrence and development of some cancers (Schwabe and Jobin, 2013; Garrett, 2015; Liu et al., 2022). With the in-depth study of lung microorganisms, we understand that the microbial diversity plays an important role in regulating the lung immune environment and maintaining the development of the immune system. In our study, especially in the analysis of co-abundance network, two phylum level bacteria were prominent, *Firmicutes* and *Proteobacteria*. Zhang et al. (2018) found that the abundance of *Firmicutes* in lung cancer patients was significantly lower and the *Bacteroidetes* were significantly higher than that in healthy people. Liu et al. (2019) showed that *Firmicutes* can convert undigested carbohydrates and proteins into acetic acid, and then provide energy for life activities. It plays an important role in the process of carbohydrate transport and metabolism, which reveals that the change of bacterial microbiome changes the energy metabolism pathway of lung cancer patients, and may further affect the progress of the disease. At the same time, more and more evidences have shown that *Proteobacteria* may cause metabolic disorders, inflammation, and even cancer. Recent studies have shown that in asthma and many lung inflammatory diseases, *Proteobacteria* expand uncontrollably, and the distribution of pulmonary microorganisms changes toward *Proteobacteria* (Hilty et al., 2010; Molyneaux et al., 2013), which further lead to the occurrence and development of LUAD. In summary, a limited but growing number of literatures indicated that the decrease in microbial diversity or increase in the abundance of taxa of *Firmicutes* and *Proteobacteria* may be related to the increased risk of lung cancer.

In our study, compared with microbial markers, the DEGs obtained by mRNA screening were more accurate in predicting patients with LUAD, and may have more obvious changes in gene expression in patients with advanced LUAD than changes in microbial profiles. Literature data show that RN7SK RNA plays an important role in the process of neurodegeneration (Santoro et al., 2016), and the loss of this small RNA will reduce the transcription of cell cycle regulators, leading to cell cycle exit and differentiation, which may affect the occurrence of cancer (Bandiera et al., 2021); Liang et al. (2022) found that SNORD17 expression was significantly up-regulated in patient tissues. It drove cancer progression by inhibiting p53 signal in hepatocellular carcinoma, and is also a potential therapeutic target for hepatocellular carcinoma. Huang R. et al. (2022) constructed acute myelocytic leukemia prognostic markers based on 14 prognostic SNORNAs and achieved a good predictive effect. Although the pathogenic mechanism of these biomarkers for patients with LUAD is still unclear, we can reasonably speculate that they promote the development of cancer.

According to the machine learning model we constructed, the predictive ability AUC of transcriptome gene expression to different stages of patients reached 0.66. However, the AUC increased to 0.70 after the addition of microbial markers. This indicated that microbial markers not only have certain predictive ability for LUAD stage, but also can make up for the lack of information of single omics. The intratumoral microbes we identified can be used as a novel predictive marker to combine with existing markers that predict the stage of lung cancer, such as proteins in the blood. The fusion of data from these different modes may further improve the performance of models that predict tumor stages and aid in the development of methods for early lung cancer screening and diagnosis.

Also, this study has some limitations. First, although we comprehensively compared the intratumoral microbiome profiles of patients with LUAD at different stages, there was no external independent validation, which limited the universal applicability of our model. Second, we only integrate microbiome and RNA expression in determining LUAD stage. Some recent studies suggested that pathological image is quite important in cancer diagnosis and prognosis prediction (Huang K. et al., 2022; Liu et al., 2022; Yang et al., 2022; Yao et al., 2022), which presents the need for integrating more types of data into our model. Third, bacteria and bacterial structures within tumors are found in cells such as tumor cells and immune cells. A number of published deconvolution based algorithms can be used to infer the relative proportions of cells from transcriptome data (Li et al., 2020). Therefore, the introduction of tumor microbiome information at single-cell resolution in the future could facilitate the understanding of tumor development mechanisms.

## 5 Conclusion

In summary, through a comprehensive analysis of patients with LUAD at different stages, we identified bacterial microbial markers related to stage, and proved that the microbiome can make up for the lack of information of other omics and assist doctors in clinical diagnosis. This study may broaden our understanding of the molecular pathogenesis of LUAD and provide new ideas for staging prediction.

## Data availability statement

The original contributions presented in the study are included in the article, further inquiries can be directed to the corresponding authors.

## Author contributions

YS: Conceptualization, Data curation, Formal analysis, Methodology, Writing – original draft. SL: Formal analysis, Writing – review & editing. DS: Conceptualization, Investigation, Supervision, Writing – review & editing. YZ: Conceptualization, Supervision, Writing – review & editing.
